# Exploring the therapeutic potential of reading: Case studies in diary-assisted reading

**DOI:** 10.3389/fpsyg.2022.1037072

**Published:** 2022-11-17

**Authors:** Kelda Green

**Affiliations:** Centre for Research into Reading, Literature and Society, University of Liverpool, Liverpool, United Kingdom

**Keywords:** bibliotherapy, reading and psychology, medical humanities, empirical studies in reading, diary-assisted reading

## Abstract

This article explores the impact that literary texts can have on real lives and considers how, as researchers, we can get closer to the authentic, first-hand experience of reading to better understand its potential therapeutic mechanisms. Three case studies, based on reading diaries kept over a period of 2 weeks and subsequent semi-structured interviews, are presented here, to demonstrate what it is that literature can do to and for us. The methodology used to collect the case studies offers a simple and replicable template both for encouraging deep reading and for capturing its resulting therapeutic and psychological outcomes. By highlighting the real experiences of readers as they encounter and engage with a complex and unfamiliar literary text, this article aims to demonstrate how empirical research methodologies can add new purpose and meaning to literary study, and equally, show how “literary thinking” can contribute to the methodologies of psychological study and the formulation of psychological therapies.

## Introduction

Rita Felski writes in *The Limits of Critique* that:

““Receptivity”… refers to our willingness to become unclosed” to a text, to allow ourselves to be marked, struck, impressed by what we read. And here the barbed wire of suspicion holds us back and hems us in, as we guard against the risk of being contaminated and animated by the words we encounter (Felski, 2015).

The challenge for researchers interested in the intersection of literature and psychology, and in the empirical study of reading, is how to create a space where readers can open themselves to the possibility of being “marked” or “struck” by a text, and to ensure that those resultant marks can be studied objectively. This article offers a replicable methodology with which we can begin to do this interdisciplinary work. Reading here is not an academic exercise or an explicit therapy. This work sits at the intersection between literary study and psychology and seeks to reinvigorate and challenge both disciplines and the toolkits that they use to do their work.

Wordsworth—the archetypal healing poet—sits at the heart of this study. There is a long tradition of readers and writers attesting to the therapeutic power of his poetry, and the reading experiment discussed here sought to test that therapeutic power on modern readers. Wordsworth’s commitment to prevent the wastage of emotional matter—most particularly grief—is important and speaks to the world’s current psychological predicament. In the aftermath of a pandemic, we are navigating concentric circles of grief and bereavement: personal, local, national, and global. How do we start to think about and speak about these experiences, if we possess an over-medicalized and quantitative language for processing grief that, night after night, distils bereavement into graphs and charts displayed on the news? In his poetry, Wordsworth offers us an alternative language for thinking about troubles and existing in our grief: not attempting to deny or alleviate its difficulty, but somehow guiding us to put that trauma to use.

The case studies presented here have grief as a common thread. There is the young man who recalls the death of his father 10 years earlier and explores—with what seems like a new syntax and vocabulary learned from his reading—the contagion of grief, passed onto him by his mother. The middle-aged woman, through her reading of the poem, is taken back to her experience of miscarriage, years earlier. And the headteacher, who abandons her default, academic mode of reading the poem, almost against her own will, to face her mother’s recent cancer diagnosis. The case studies offer a glimpse of the helpful patterns of thought that reading may open up to us, and the unexpected moments of empathy that they spark, between people and equally, between past and present versions of the same person. Key findings that emerged from the reading diaries of the group include:

1. Readers begin to shift out of default modes of thinking, reassessing their own approach to the poem and adjusting their way of thinking about it as they progress.
2. Readers are able to explore the wider span of their whole lives, quickly getting into the thick of unconscious or unexpected areas of thinking.
3. Readers seem to quickly access unprocessed traumas and begin—with the guiding influence of the poem—to put that trauma to some use within their own minds.

## Methods

Twenty adults were recruited to take part in a reading study through advertisements placed across The University of Liverpool campus and in public libraries in Liverpool and London. The only criterion for inclusion was that participants were fluent English speakers. Recruits were aged between 19 and 71 years and had varied relationships with reading: some were avid readers who had studied, or even taught literature, and others had little or no prior experience of tackling a poem.

Participants were divided into two groups, with care taken to ensure that a similar range of ages and educational experiences, and genders were represented in both groups. The first 10 participants were asked to spend 30 min per day for a period of 2 weeks writing about anything that was important to them. The second group of participants was given a plain notebook containing the text of William Wordsworth’s poem “The Ruined Cottage.” The 1,009-line poem was split into 14 sections of approximately 70 words each. Participants were asked to spend 30 min each day for a period of 2 weeks reading one section of the poem and writing about anything that they felt to be interesting or important to them. Following the completion of their task, readers were invited to semi-structured interviews. Interviews lasted between 1 and 2 h and began with a series of preset questions to understand how the participant had tackled their task and the effect, if any, that it had had on them. Particularly, salient diary entries were pre-selected by the interviewer in advance of the interview to be re-read and discussed; however, it was through open-ended conversation and reflection during the interviews themselves that both the interviewer and participant selected passages together to re-read and think through again out loud. The purpose of the interview was to encourage deeper reflection and further specificity of thought from participants. In the interviews, as throughout this study, my methodological model is practical criticism—the technique of literary analysis that centers on the close and careful reading of the words on the page (Richards, 1930). The diaries and interview transcripts were cross-checked by a group of three psychologists trained in qualitative analysis to build consensus around themes emerging from the data. Each member of the group coded a set of transcripts before coming together for a series of discussions to explore commonalities and differences in perspectives.

The methodology of diary-assisted reading was developed to encourage participants to establish a structured reading and/or writing routine and to engage in daily focused reflection for a period of 2 weeks. It was important that the structure of the task guided participants toward a slower pace of reading. By slowing down, it was hoped that readers—both those with little experience of reading poetry and those with prior literary training—would be able to break away from any learned behaviors or preconceptions connected to reading. The aim throughout was to capture reading in action and to avoid—as far as possible—reductive summary mode. It was important to create an opportunity for readers to respond instinctively and honestly to the text, to let their guard down, and to read and write with vulnerability and sensitivity. The process of shedding preconceptions and moving away from too-easy default modes was a noted feature of many of the groups who were writing in response to the poem, while it was absent from all those participants who were simply writing a daily diary.

The notebooks given to participants contained plain, unlined paper. Each book was tied with a ribbon and the passages of poetry were roughly cut out and glued onto the pages. A handwritten note was included with instructions at the start of each diary. These small, seemingly inconsequential choices were deliberate. They intended to disarm readers and mitigate the perceived “threat” of the text—articulated by many of the group, who although had voluntarily signed up to take part, initially spoke of their fear of poetry or of being intimidated by past experiences of academic reading and writing. The task had to have none of the look and feel of an academic assignment. Equally, it was important that the methodology contained no explicit reference or echo of a therapeutic task. Participants should not feel that they were undertaking a version of self-help or therapy. Instead, any therapeutic mechanism should be accidental and spontaneous.

The task was designed to bring readers into contact with a Wordsworthian sense of the therapeutic—which is never explicit—but instead related to slowing down and staying within a mental and physical space. The poem “The Ruined Cottage” which was selected for use in this task tells the story of Margaret—a woman abandoned by her husband and desperately awaiting his return. The story is told by a pedlar who once knew Margaret, remembering her life and tragic decline as he sits beside her now ruined former home. It is a poem that places us quietly within the small details of Margaret’s life and contains one of the best examples of Wordsworth’s version of a kind of therapeutic transmutation. As the Victorian critic Leslie Stephen—who personally attested to the therapeutic benefits of reading Wordsworth—wrote of the poet’s commitment to the transmutation of suffering: “Wordsworth’s favorite lesson is the possibility of turning grief and disappointment into account. He teaches in many forms the necessity of “transmuting” sorrow into strength […] the waste of sorrow is one of the most lamentable forms of waste” (Stephen, 1892).

This article does not propose to set out a detailed breakdown of the results of this study or to provide a comparison of the diaries produced by the two groups of participants—which can be found elsewhere (Green, 2020).^1^ Instead, I have been purposefully selective with my choice of case studies to begin to draw out thinking about the potential specific value of literary texts in helping humans to think through their experiences of grief. My intention here is not to force a hypothesis out of what is clearly limited data, but rather to begin to show how a literary approach to methodology and psychology more generally may have potential, particularly in relation to how people understand grief. There are obvious limitations to this approach and to the general methodology that I have selected. The presentation of the notebooks and the structure of the tasks may have led to participants feeling obligated to perform in a certain manner. They may have felt a pressure to self-edit their writing, in the knowledge that it would be read and analyzed. A desire to please or perform well may have extended to the interviews, where face to face with the researcher, participants may have felt even more inclined to self-edit or to tell me what I wanted to hear. The small number of participants recruited for the study and the method of recruitment—through libraries and a university—could cause concern and may have limited the diversity of the group. The act of daily writing, particularly for those writing in parallel to reading a potentially emotionally triggering text may lead to potential risks for vulnerable individuals and a power imbalance between researcher and participant. These issues were considered carefully during the study design and in part mitigated by the attempts previously described to uncouple the study from any academic or therapeutic associations. Ethical approval was obtained from The University of Liverpool and all participants provided informed consent before beginning the study. Participants could withdraw at any time. One person did withhold their final diary and agreed only to be interviewed about their experience of the task, feeling that sharing their writing would expose an element of themselves that they were not willing to.

## Results

### Case study one: Grief is contagious

Michael is a 26 year old waiter from London. He left school aged 16 after completing his GCSEs and has a keen interest in art and design. He has no memory of reading poetry outside of school and was initially hesitant about taking part in the study, commenting that he “felt anxious about doing it wrong,” a statement which legitimizes some of the concerns that I had during the study design about the perceived “threat” of an academic task—which persisted for some participants despite attempts to reduce this anxiety. In his first two diary entries, Michael wrote long, descriptive summaries of the extract that he had read that day. However, on the third day of the task, Michael abruptly altered his method. After reading the day’s passage, he wrote:

Stop thinking/being so analytical—feelings/instincts are real and important. I feel I must take this on board. (Day Three)

From this point on, Michael stopped paraphrasing and instead began to write about what the reading was making him feel each day. Michael went on to write about deeply personal topics. In particular, the grief that he and his mother had experienced after his father had died, 10 years earlier, when Michael was 16 years old.

On day 12, Michael read a passage of the poem which tells of Margaret’s spiraling decline into despair and her subsequent neglect of her young child. The passage includes the lines:

Her infant babe

Had from his Mother caught the trick of grief,

And signed among its playthings (Wordsworth, 2007, I, ll.868–70).

In response to this passage, Michael wrote:

Grief is contagious. It seems strange to me that she would seem so affected by her husband’s grief yet so unaffected by the death of her child. Or I suppose her husband’s death had so affected her that the remainder of him (her child) was a burden. Makes me jealous of that kind of love. You want to scream at her “pull yourself together” which makes me feel as though I’m unsympathetic and dead inside myself. (Day Twelve)

Here Michael is writing about both Margaret and his own mother. He is thinking simultaneously about Margaret’s husband and his own father and about Margaret’s child and his own self. The poem appears to be allowing him to draw closer to his own parallel experiences and to think and say what would be difficult—if not impossible—for him to say to his mother, “Pull yourself together” or “I’m jealous of that kind of love.”

During his interview Michael spoke in more detail about how reading the poem had led him to think about grief in a way that he might otherwise have avoided:

*Michael:* The poem made me think of myself as someone who may have been affected by grief unknowingly.

*Interviewer:* What do you mean by that?

*Michael:* Because I have only become a little more tolerant of talking about my own father in the last I would say year or so. Because I never really liked talking about it or anything else and I can’t say I was consciously affected by it at the time. It was not… because in the poem it talks about this old woman, and I very much saw my own mum in it. Because she is not the same woman… even now. She is nowhere near what she was. And that was what kind of… I can see how I’m probably a bit more severe in my own nature because she was so miserable. Because I was quite happy go lucky really before that… you know… because I don’t like saying it was… because I can’t say I ever got on with him, I can’t say I ever really liked him, I can’t say it was a big loss personally. It was just that thing of how it affected my mother and subsequently how that affected me. Because that was in the poem, I think I just couldn’t not write what I wrote. (Interview)

The poem has caused Michael to begin to think about the ways in which grief was transferred between his mother and his own teenage self after the death of his father. This new sense of previously unrealized feeling is articulated here in the silences between words as well as in the words themselves. All of the things that Michael feels he “can’t say” pile up here, and yet, it is as if with the help of the poem and in some way shielded by the syntax of the negative, he *is* here able to say the unsayable. This is a small example of a kind of creative inarticulacy that can prove to be therapeutic.

It was moments of personal breakthrough such as this—triggered by immersive, contemplative reading—that this experiment sought to explore, document and analyze. They demonstrate how poetry can guide readers into areas of serious emotional depth and provide them with a language for thinking that helps previously unformed thoughts to surface. Michael has for many years avoided the subject of his father’s death, but the poem meant that “I just couldn’t not write what I wrote.” The double negative form is itself borne out of the literary language that the reader has absorbed, a language that can help us to take hold of those things that we so often do not have the words for, or cannot bear to say.

### Case study two: The useless fragment of a wooden bowl

Simone is a 45-year-old Marketing Manager from Liverpool. Unlike many of the participants who quickly began to disclose and explore memories of and feelings about their own lives in their diaries, Simone’s submission appeared guarded and focused almost exclusively on her interpretation of the poem, with little allusion to her personal life. However, on day four of the task, there was a sudden surfacing of private trauma and loss. On the page, Simone had highlighted the following lines:

Beside yon spring I stood,

And eyed its waters till we seemed to feel

One sadness, they and I.

…

Stooping down to drink,

Upon the slimy foot-stone I espied

The useless fragment of a wooden bowl,

Green with the moss of years, and subject only

To the soft handling of the elements:

There let it lie – how foolish are such thoughts!

(Wordsworth, 2007, I, ll.484–496)

In response to these lines, she wrote:

Soon after I first got married, I quickly became pregnant. But ten weeks later I had a miscarriage. The baby had not survived longer than six or seven weeks, but the bleeding didn’t start until the night before my first hospital appointment was due to take place. The doctor said that she would do a scan. She watched on a screen that was angled away from me, but still within view of my husband. She doesn’t yet know that he is a doctor too, and he can read the shapes of the ultrasound. So in turn, I watch his eyes as he searches. And I understand what is coming, before the doctor says: “I’m struggling to find an embryo.” There is a long, empty silence as she continues to search. Scanning the ocean floor, looking for a sunken wreck. “There is no blood supply,” she says. “I’m very sorry.” Then she tilts the screen for me to see the black hole inside of me. Lit up all around with the neon glow of blood, a clear circle of blackness at the center. It looked like an image of outer space, taken by a telescope. Why am I writing about this here? It feels inevitable. There is a quietness to death. We are so precarious and that image of my absent baby, that black circle on the screen, was the point at which I felt that precariousness most acutely. There is something about the line in the poem about the “useless fragment of a wooden bowl,” I can’t help thinking of that scan. The emptiness of it. The poem takes me back to that exact moment in my life. Maybe someone else wouldn’t understand the connection. But I feel as I’m reading that the poem understands the connection.

“There let it lie” the poem says. Okay, I will try. (Day Four)

As Simone writes about this deeply personal experience of grief, she flips into the present tense, “she doesn’t yet know that he is a doctor too,” “I watch his eyes.” It is as if the poem has helped to transport her back into that specific time and place of trauma. Within that experience, Simone is casting about for an image—some form of language—which will reflect what is happening to her; the doctor is searching for a “sunken wreck” on the ultrasound, the image of her empty womb is like something from “outer space.” This is both an epic loss and simultaneously a moment of small, near silent, domestic tragedy. Fourteen words are spoken by the doctor—each of them quoted years later by Simone—as if seared in her memory. Both husband and wife are silent, communicating only through their watchful eyes. As in the poem, words are not required for the transfer of feeling and of understanding to take place, “I stood/And eyed it’s waters till we seemed to feel/One sadness, they and I.” The image of the “useless fragment of a wooden bowl,” that Simone—like the pedlar in the poem—has stumbled upon here, powerfully addresses that need within her for an image that corresponds with her feelings. She writes of the poem “understanding the connection” and a level of trust emerges between the reader here and the text itself—borne out of the sense of correspondence that she feels.

Just as Michael had said, “I couldn’t not write what I wrote,” here Simone writes of the inevitability of addressing her miscarriage within the diary. The poem has led both readers into the thick of their emotional lives and turned what—in regular default mode would be difficult if not impossible to speak of—into an inevitability. In the final sentence of her diary entry for the day, Simone quotes from the poem again, “There let it lie.” Held within these four words is a sense of Wordsworth’s own hard learnt lesson on grief. It cannot be gotten rid of, left behind, fixed, or perhaps even ever fully understood. Instead, this damaged, broken, painful thing that we hold, should be set down, given its place and “let” to exist there.

### Case study three: It was a plot of garden ground run wild

Samantha is the head teacher of a secondary school in the South of England. She teaches English and has studied English Literature to postgraduate level. She is familiar with the poetry of Wordsworth, and in her initial diary entries she frequently referred to what she had formerly learned about Wordsworth at university:

I think that the description of the sun alongside the ‘brooding clouds’ description, with the impact of both together on the land, creates a metaphor for the world that I remember from my uni days that this poet inhabited. (Day One).

However, over the course of the task, Samantha appeared to move away from her default academic mode and shifted to a more personal way of reading and writing.

On day 7—half way through the exercise—Samantha reads a description of Margaret’s overgrown garden:

It was a plot

Of garden ground run wild, its matted weeds

Mark’d with the steps of those, whom, as they passed,

The gooseberry trees that shot in long lank slips,

Or currants, hanging from their leafless stems,

In scanty strings, had tempted to o’erleap

The broken wall.

[…]

“I see around me here

Things which you cannot see: we die, my Friend,

Nor we alone, but that which each man loved

And prized in his peculiar nook of earth

Dies with him, or is changed; and very soon

Even of the good is no memorial left.”

(Wordsworth, 2007, I,ll. 452–74)

In response, Samantha no longer writes as a teacher or a student, she instead begins to react to the poem as a daughter coming to terms with her mother’s recent cancer diagnosis:

I had quite a shock then, reading a description of the overgrown garden, with words like “matted,” “leafless,” “long lank,” “scanty strings” leading then into the man saying what he says about death, and all that we “prize” changing and going with us when we die. I wasn’t wanting to think about that, as mum prepares for her op. I’m thinking about the gentle pauses, her lovely garden staying just fine at home for her while dad waits for her to recover and all proceeding forward as it should. I am also a little repelled by the idea that there is “no memorial left” even of the good. (Day Seven)

Samantha appears to find the passage uncomfortable to read. Being faced with an image of an imagined future that she is not ready to accept might become a reality for her is a shock to Samantha and it is this sense of shock that forces her to drop her previous default mode of academic detachment. In this case, her requoting of words from the text is a good thing to come out of her student/teacher experience as it creates a more focused, live reading of the poem. Two weeks later at her interview, I read aloud this same passage of poetry about Margaret’s overgrown garden and Samantha spoke again about the fear that it had initially triggered in her:

Not wanting anything to alter or adjust at all and how like the processes of nature happen anyway no matter what you do and that is quite scary. I’ve found that, I like that, I really love that passage and I really connected with that passage but at the same time it showed a really powerful advancing of time and nature that is a little bit scary, especially if you are faced with illness at a particular point. The garden itself reminded me of where we lived as kids, they or we had a big garden and I imagine it like now, I imagine it like that, even though they don’t live there now. I imagine that if they did, that if mum, I mean mum’s getting better, but if she wasn’t, there would be that sense of that happening there, and how sad that is. I think that that is a really overwhelmingly sad passage, really powerfully, really powerfully. And how when you go and revisit places, I don’t like going and revisiting places particularly because I often find that the experience that you had is altered and not always in a good way. I think that is why that spoke to me as well really strongly in terms of mum’s illness, because I don’t like going back to where we lived as children in case it has all gone wrong. (Interview)

Rereading this passage of the poem during the interview seemed to help Samantha to quickly get back into the emotions that the text had initially triggered in her. As soon as I had finished reading the passage, she began to speak—not in fully formed sentences—but urgently mid-sentence, keen to articulate, however roughly, the mixture of feelings and memories that the poetry had activated. Reading seems to have become an unexpectedly personal and imaginative process for Samantha, and her case study hints at how reading can perhaps give us a means of imaginatively restoring a place that cannot be returned to in reality.

## Discussion

Key findings illustrated in part through the case studies presented here were:

1. Readers begin to shift out of default modes of thinking, reassessing their own approach to the poem and adjusting their way of thinking about it as they progress.

It is perhaps in matters of grief that our default modes of thinking about and processing the world show themselves to be most inadequate. Grief demands more from language, although not more in terms of articulacy or even vocabulary, it demands a more nuanced syntax that can accommodate inarticulacy and silence within it. As Michael clearly showed in his early change in approach to reading the poem, his automatic default of overexplaining and describing did not serve him. In realizing this, he was able to adapt and get more out of the poem and himself as a result.

2. Readers are able to explore the wider span of their whole lives, quickly getting into the thick of unconscious or unexpected areas of thinking.

The poem appeared to act as a guide to readers, drawing them back into the thick of their emotional pasts in a way that was unexpected for many and perhaps also uncharacteristic. The poem appeared to give more to the readers than they had expected, once those default barriers had been disarmed, and as such they in turn were able to offer more back in the process of reading.

3. Readers seem to quickly access unprocessed traumas, and begin—with the guiding influence of the poem—to put that trauma to some use within their own minds.

In small ways, readers appeared to hit upon new truths or previously unrealized understandings of their past experiences, and while this sometimes involved returning to places of intense personal sorrow for them, by returning, staying within and in some way reconfiguring that sorrow, through the supportive correspondence of the poem, they were able to achieve some sort of therapeutic breakthrough.

## Data availability statement

The original contributions presented in this study are included in the article/supplementary material, further inquiries can be directed to the corresponding author.

## Ethics statement

The studies involving human participants were reviewed and approved by Institute of Psychology, Health and Society, the University of Liverpool. The patients/participants provided their written informed consent to participate in this study. Written informed consent was obtained from the individual (s) for the publication of any potentially identifiable images or data included in this article.

## Author contributions

The author confirms being the sole contributor of this work and has approved it for publication.

## Footnotes

1 The texts produced by group two were expansive in their scope, drew heavily on the participants memories and early childhoods and quickly got into the thick of emotional troubles that participants may have experienced. Conversely, diaries produced by those in group one focused exclusively on the present. While those in group two demonstrated the capacity to shift perspective over the course of the 2-week period, adapting their approach to the task and casting off default assumptions, there was no evidence of this in group one.

## References

[B1] Felski R. (2015). *The Limits of Critique.* Chicago, IL: University of Chicago Press. 10.7208/chicago/9780226294179.001.0001

[B2] Green K. (2020). *Rethinking Therapeutic Reading.* London: Anthem. 10.2307/j.ctvsn3p9x

[B3] Richards I. A. (1930). *Practical criticism.* London: Kegan Paul.

[B4] Stephen L. (1892). “Wordsworth’s Ethics,” in *Hours in a Library*, (London: Smith Elder).

[B5] Wordsworth W. (2007) *The Excursion.* Ithaca, NY: Cornell University Press.

